# Long‐term outcomes of epilepsy surgery: A 25‐year experience from a tertiary referral center

**DOI:** 10.1002/epd2.70101

**Published:** 2025-09-06

**Authors:** Marco Almeida, Francisco Barros, Inês Cunha, Ana Brás, Rute Teotónio, Conceição Bento, Francisco Sales

**Affiliations:** ^1^ Referral Center for Refractory Epilepsy Epilepsy Surgery Program Group – ULS Coimbra Coimbra Portugal; ^2^ European Reference Network EpiCARE Lisbon Portugal

**Keywords:** ECOG, epilepsy, mesial sclerosis, refractory epilepsy

## Abstract

**Objective:**

Despite pharmacological advances in epilepsy treatment, one‐third of patients remain pharmacoresistant and may require surgery. Despite extensive literature on epilepsy surgery, studies with follow‐ups longer than 5 years are rare. Our goal was to analyze the outcomes of patients undergoing epilepsy surgery at our center, with a minimum follow‐up of 15 years.

**Methods:**

This was a retrospective study of prospectively collected data. We used the Engel classification to assess seizure freedom, performed univariate descriptive analysis of the variables of interest, and applied appropriate correlation tests for nominal and categorical variables, with statistical significance set at 0.05.

**Results:**

We included 160 patients with a minimum follow‐up of 15 years. A total of 105 (70%) patients underwent resective surgeries, the most common being lesionectomy (46.7%), followed by anterior temporal lobectomy with amygdalectomy (21.9%). Among resective surgeries, 73.6% used intraoperative ECOG. Most surgeries were in the temporal lobe (68.8%), and mesial sclerosis was the most frequent etiology (33.8%), followed by long‐term epilepsy‐associated tumors (LEAT) (25.6%). Seizure freedom at 15 years was achieved by 57.5% of patients, and most of the remaining patients (63.2%) had rare disabling seizures. The majority (65%) discontinued at least one ASM. Temporal surgeries (*χ* ^2^(1) = 8.444, *p* < 0.05), left‐sided surgeries (*χ* ^2^(1) = 6.436, *p* = 0.04), mesial sclerosis (*χ* ^2^(1) = 50.870, *p* = 0.024), and the use of intraoperative ECOG (*χ* ^2^(1) = 23.235, *p* < 0.001) were associated with a better prognosis. No differences in outcome were found between the different temporal lobe surgeries (Fisher's exact test value = 0.859, *p* = 0.659).

**Significance:**

Appropriate referral to a refractory epilepsy center permits a multidisciplinary approach that can result in long‐term seizure freedom for most patients undergoing surgery, especially for left‐temporal lobe surgeries performed with the aid of intraoperative monitoring techniques.

**Key points**

- In this sample, 57.5% of patients remained free of disabling seizures (Engel Class I) at 15 years of follow‐up after epilepsy surgery.
- Patients with temporal lesions were more likely to achieve seizure freedom when compared to patients with extra‐temporal lesions.
- Lesion laterality and etiology were also associated with outcomes, with left‐sided lesions and mesial sclerosis associated with the best results.
- In our sample, the use of intraoperative ECOG was also associated with better outcomes.
- In this study, we found no statistical differences between different types of temporal lobe epilepsy surgery.

## 1 INTRODUCTION

Despite recent pharmacological advances in the treatment of epilepsy and considerable variation between studies, it is estimated that more than 30% of patients are classified as drug‐resistant (not responding to at least two appropriate medications for seizure control with tolerable side effects).^1^, ^2^ Surgery has emerged as a solution for some of these patients with both temporal and extratemporal epilepsy. As highlighted by multiple reviews, most epilepsy surgery outcome studies report follow‐up durations of 1 to 3 years, with relatively few extending beyond 5 years, underscoring the scarcity of long‐term postoperative data in the literature.^3^, ^4^, ^5^ To our knowledge, the longest published follow‐up after epilepsy surgery is reported by Mohammed et al.,^6^ with a mean duration of 26.5 years (range 21–42 years). However, this cohort underwent surgery between 1967 and 1990, a period characterized by older surgical techniques, limited access to advanced imaging modalities such as MRI and SPECT, and less comprehensive preoperative evaluations, including neuropsychological assessment. In contrast, our study reflects a more modern era of epilepsy surgery, with patient selection guided by multimodal imaging, detailed neuropsychological profiling, and refined surgical techniques, offering a more complete and contemporary perspective on long‐term outcomes. We aimed to report the surgical outcomes of patients operated on at our 25 years' experience referral center. An option was made to perform an analysis including only patients who underwent resective or disconnective surgeries, and excluding neuromodulation device implantation surgeries, in order to facilitate comparison with prior studies using similar inclusion criteria.

## 2 METHODS

### 2.1 Study design, patient selection, and collected data

We designed a retrospective longitudinal observational study conducted at the Refractory Epilepsy Center of Unidade Local de Saúde de Coimbra, in Portugal. After informed consent was obtained, we included all patients who underwent resective and disconnective epilepsy surgery between January 1997 and December 2009 and evaluated their postoperative outcomes. We excluded the group of patients with neurostimulation devices. Patients with insufficient data or who died before reaching 15 years of follow‐up were excluded from the study.

We collected demographic information such as age at the time of surgery, gender, seizure frequency before and after surgery, number of anti‐seizure medications (ASM) before and after surgery, epilepsy etiology, affected brain side and lobe, as well as date and type of the surgery. Based on information gathered from clinical records and phone calls, we classified the surgical outcome using the Engel scale of outcome. In this classification, the seizure outcome was sorted into four classes and defined as follows: Class I—free of disabling seizures; Class II—rare disabling seizures; Class III—worthwhile improvement; and Class IV—no worthwhile improvement.^7^ The surgical outcome was determined based on the most recent follow‐up data and compared to preoperative data or information collected via phone calls with patients. All data were collected in January 2025, ensuring a minimum of 15 years between the last surgery performed by the end of 2009 and the most recent follow‐up.

### 2.2 Statistical analysis

Data was analyzed using SPSS Statistics for Windows, Version 23.0 (IBM SPSS Statistics for Windows, Version 23.0. Armonk, NY: IBM Corp). Several factors related to the seizure‐free (SF) outcome were analyzed. First, we conducted a univariate descriptive analysis of the variables of interest to characterize the sample. Continuous variables are expressed as mean ± standard deviation. We applied the Shapiro–Wilk test to determine whether the continuous variables followed a normal distribution and used the parametric Pearson test or the non‐parametric Spearman test to find correlations accordingly. The *t*‐test was used for continuous variables, while the Chi‐square test and Fisher's exact test were applied to categorical variables. Given the hypothesis‐driven nature of the variables and the limited number of comparisons, a correction for multiple testing (e.g., Bonferroni) was not applied. A statistical significance level was set at 0.05.

## 3 RESULTS

**FIGURE 1:**
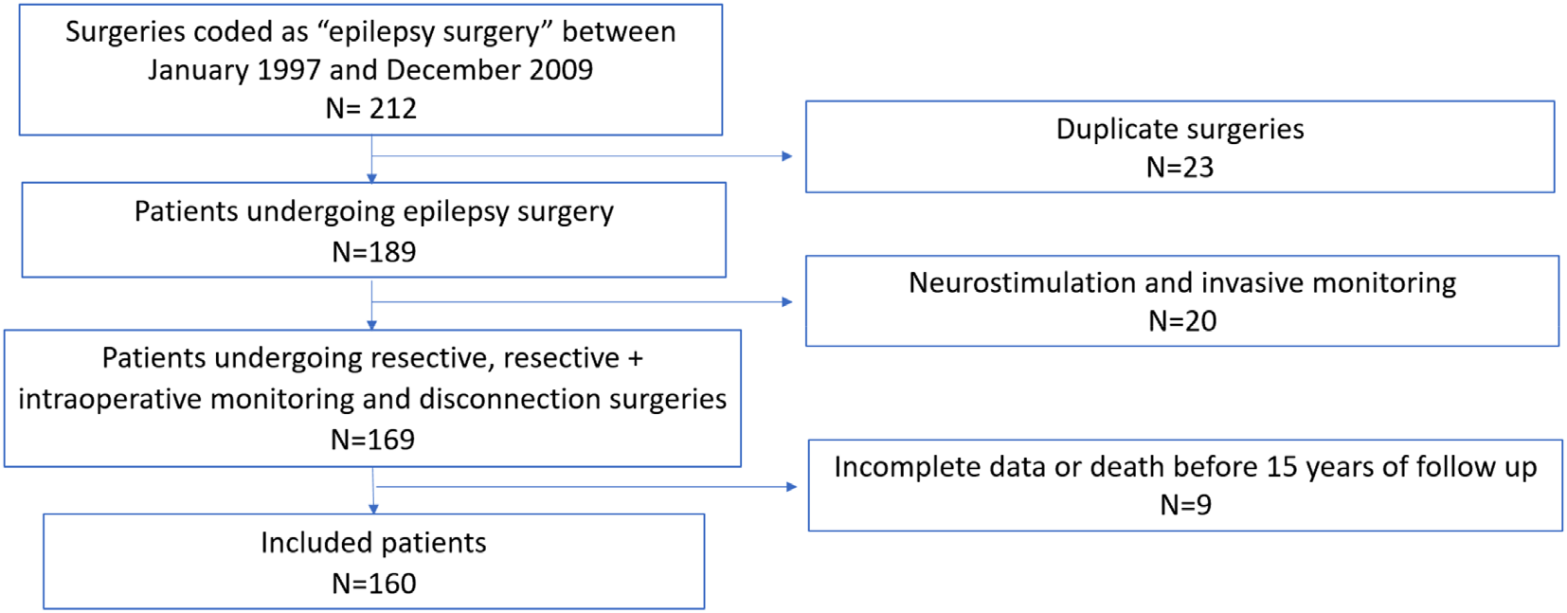
Study flowchart of patient selection.

**FIGURE 2:**
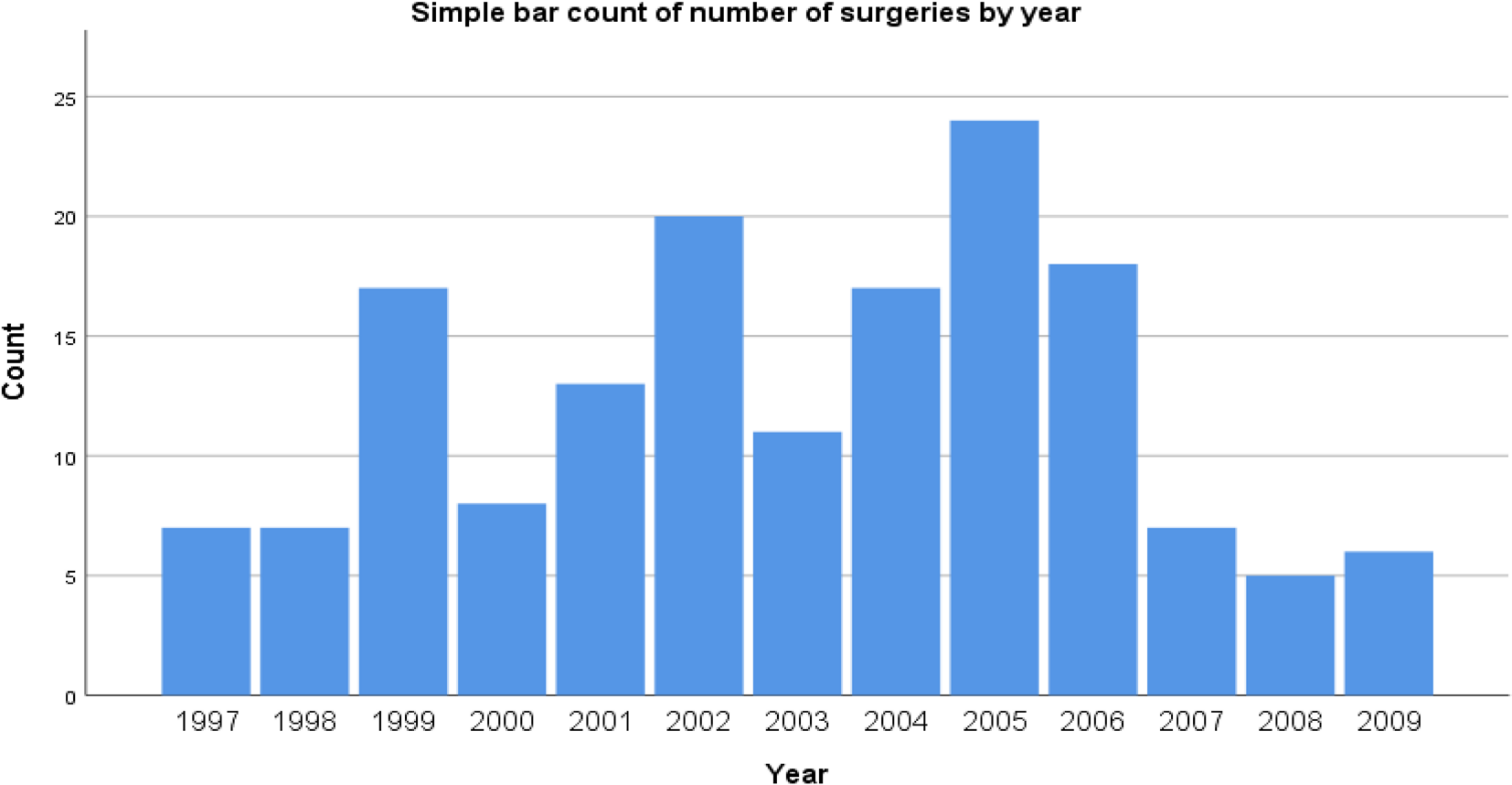
Bar count of surgeries by year.

**FIGURE 3:**
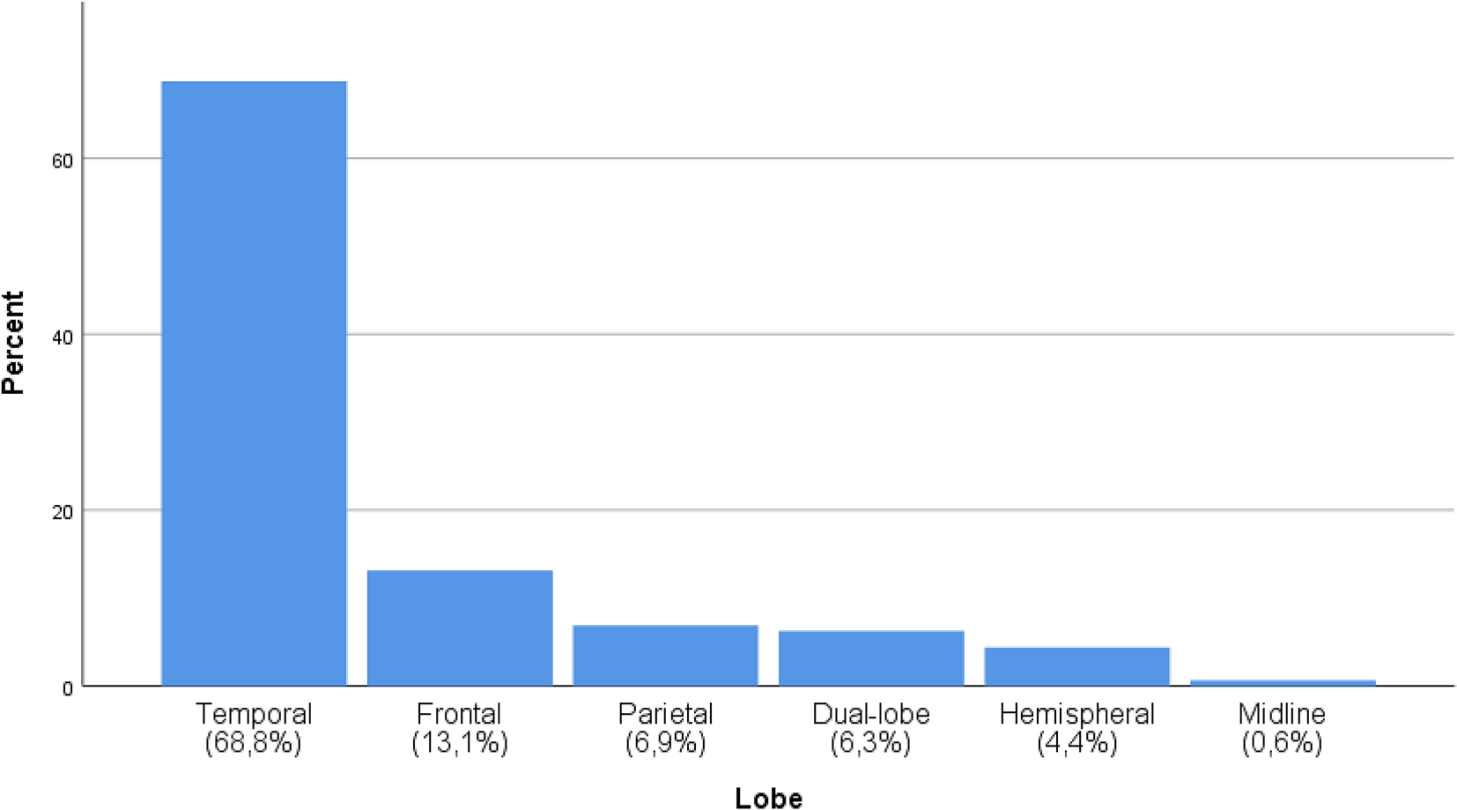
Bar count of surgeries by cerebral lobes.

From an initial number of 212 epilepsy surgeries within the selected period, 189 patients were selected and, after applying the exclusion criteria, 160 patients were included (Figure 1). The mean age was 31.94 years (SD 12.76), ranging from 3 to 61 years. The gender distribution was balanced, with 50.6% being female. The average follow‐up time in our sample was 17.22 years (SD 3.61). At the time of publication of these data, 26 patients (16.3%) are still being actively followed in our institution. Additionally, 9 patients (5.6%) who underwent surgery during the study period died after completing more than 15 years of follow‐up, and they were therefore included in the analysis. None of these deaths were attributed to SUDEP (sudden unexpected death in epilepsy). The surgeries were performed between 1997 and 2009, with 2005 being the most productive year, accounting for 23 surgeries (14.4%) (Figure 2). Most of the surgeries performed were resective, totaling 105 procedures (70%). The three most common types of surgeries were lesionectomy, with 70 cases (46.7%), followed by anterior temporal lobectomy with amygdalohippocampectomy in 35 patients (21.9%), and partial anterior temporal lobectomy plus amygdalectomy in 13 patients (8.1%). A total of 109 procedures (68%) were performed with intraoperative electrocorticography (ECOG), and this percentage increases to 73.6% when disconnective surgeries are excluded (Table 1). Of the procedures performed, 110 were in the temporal lobe (68.8%). We considered procedures performed in other lobes or with dual intervention in two lobes, even if one of them was temporal, as extra‐temporal. The second most commonly operated lobe was the frontal lobe (Figure 3). Most surgeries were conducted in the left hemisphere, totaling 88 procedures (55%), followed by 68 procedures (42.5%) in the right hemisphere and 4 procedures (2.5%) with bilateral or midline interventions. Regarding the etiology of refractory epilepsy, mesial sclerosis stands out in 54 patients (33.8%), followed by long‐term epilepsy‐associated tumors (LEAT) in 41 patients (25.6%). The complete list of identified etiologies by cerebral lobe is presented in Table 2. Similar to other authors, we chose to group low‐grade astrocytic or glioneuronal lineage tumors as LEATs for classification purposes.^8^, ^9^

**TABLE 1:** Absolute count and percent of surgeries performed between 1997 and 2009 and relative percent of seizure‐free patients.

	Frequency	Group percent	Total percent	Engel I percent
*Resective surgery without ECOG (n = 39)*				
Lesionectomy	13	33.3	8.1	54
Anterior temporal lobectomy + amydgalohyppocampectomy	8	20.5	5	63
Partial anterior temporal lobectomy + amygdalectomy	3	7.7	1.9	67
Amygdalohyppocampectomy	2	5.1	1.3	50
Temporal lobectomy	3	7.7	1.9	67
Topectomy	2	5.1	1.3	50
Partial frontal lobectomy	1	2.6	0.6	0
Partial amygdalohyppocampectomy	1	2.6	0.6	100
Hyppocampectomy	1	2.6	0.6	100
Frontal lobectomy	1	2.6	0.6	0
Other dual procedures	4	10.3	2.5	50
*Resective surgery with ECOG (n = 109)*				
Lesionectomy	57	52.3	35.6	56
Anterior temporal lobectomy + amydgalohyppocampectomy	27	24.8	16.9	63
Partial anterior temporal lobectomy + amygdalectomy	10	9.2	6.3	60
Amygdalohyppocampectomy	12	11	7.5	58
Topectomy	3	2.8	1.8	67
*Disconnection surgery (n = 12)*				
Hemispherotomy	6	50.0	3.8	67
Hemispherectomy	3	25.0	1.9	67
Callosotomy	2	16.7	1.3	50
Frontal disconnection	1	8.3	0.6	100

Abbreviation: ECOG, electrocorticography.

**TABLE 2:** Etiology of epilepsy in the sample and relative percent of seizure‐free patients.

Lobe	Frequency	Percent	Engel I percent
*Temporal*	110	68.8	62.7
Mesial sclerosis	54	33.8	63
LEAT	30	18.8	56.7
Dual pathology	9	5.7	44.4
Cavernoma	7	4.4	57.1
Cortical dysplasia	3	1.9	33.3
Glioma (high grade)	2	1.2	50
NF1	2	1.3	50
Gliosis	1	0.6	100
Herpetic encephalopathy sequelae	1	0.6	100
Non classified tumor	1	0.6	100
*Frontal*	21	13.1	47.6
Cortical dysplasia	6	3.8	33.3
LEAT	5	3.1	60
Vascular	3	1.9	33.3
Cortical malformation	3	1.9	66.7
Meningioma	2	1.3	50
Cavernoma	1	0.6	100
Glioma (high grade)	1	0.6	100
*Parietal*	11	6.9	54.5
Cavernoma	4	2.5	50
LEAT	3	1.9	66.7
Meningioma	2	1.3	50
Vascular	1	0.6	0
Non classified tumor	1	0.6	100
*Dual‐lobe*	10	6.3	50
LEAT	3	1.9	66.7
High grade glioma	2	1.3	50
Astrocytoma	1	0.6	100
Cavernoma	1	0.6	100
Cortical dysplasia	1	0.6	0
Dual‐pathology	1	0.6	0
Vascular	1	0.6	0
*Hemisphere*	7	4.4	71.4
Cortical malformation	3	1.9	66.7
Hemimegalencephaly	2	1.3	50
Rasmussen syndrome	2	1.3	50
*Midline*	1	0.6	100
Hamartoma	1	0.6	100

Regarding seizure frequency before surgery, 50 out of 160 patients (31.3%) experienced monthly seizures, while 44 patients (27.5%) had seasonal seizures, and 43 patients (26.9%) had weekly seizures. The Engel classification for surgical outcomes was applied, identifying 92 patients (57.5%) as seizure‐free (Class I). A total of 104 patients (65%) discontinued at least one ASM after surgery, and 40 patients (25%) maintained the same ASM but reduced the dose of at least one medication (Table 3).

**TABLE 3:** Characteristics of epilepsy in the patients of the sample.

Engel score	Frequency	Percent
*Class I*		
I‐A	50	31.3
I‐B	24	15
I‐C	18	11.3
*Class II*		
II‐A	3	1.9
II‐B	33	20.6
II‐C	6	3.8
II‐D	1	0.6
*Class III*		
III‐A	10	6.3
III‐B	7	4.4
III‐C	4	2.5
*Class IV*		
IV‐B	4	2.5
*Frequency of seizures before surgery*		
Daily	8	5
Weekly	43	26.9
Monthly	50	31.3
Seasonal	44	27.5
Yearly	15	9.4
*Number of ASM*		
Same ASM in the same dose	9	5.6
Reduced dose of at least one ASM	40	25
Discontinued at least one ASM	104	65
Totally discontinued ASM	7	4.4

Abbreviation: ASM, anti‐seizure medication.

No associations were found between gender and seizure outcome (*χ* ^2^(1) = 0.623, *p* = 0.430) and the mean age of the seizure‐free group (*M* = 31.91, SD = 13.00) was not significantly different from that of the non‐seizure‐free group (*M* = 31.99, SD = 12.52), with a *p*‐value of 0.971. We investigated possible associations between the localization of cerebral lesions (temporal vs. extra‐temporal) and seizure outcomes (seizure‐free vs. non‐seizure‐free) using a Chi‐Square test, whose statistic was *χ* ^2^(1) = 8.444, *p* < 0.05, indicating a significant association between lesion localization and seizure outcome. The analysis showed that the observed frequency of seizure‐free outcomes was higher than expected for temporal lesions and lower than expected for extra‐temporal lesions, suggesting that patients with temporal lesions are more likely to achieve seizure freedom when compared to the extra‐temporal group. The same test was conducted to analyze associations between each cerebral lobe and seizure outcomes. Not surprisingly, a significant association between the cerebral lobe involved and seizure outcome was identified (*χ* ^2^(1) = 14.390, *p* < 0.013). When analyzing the expected frequencies for seizure‐free outcomes, the results showed that the observed frequencies were as expected in most cases, with notable deviations for the temporal and frontal lobes, where the observed frequencies of seizure‐free outcomes were higher than expected. This trend suggests that both temporal and frontal lesions are associated with more favorable seizure outcomes after surgery.

We found an association between lesion laterality and seizure outcome (*χ* ^2^(1) = 6.436, *p* = 0.04). The frequency of seizure‐free outcomes was higher than expected for left‐sided lesions and lower than expected for right‐sided lesions, suggesting a greater likelihood of achieving seizure freedom if the lesion is on the left side. Bilateral or midline lesions had observed frequencies similar to the expected frequencies. In our sample, there was also an association between the type of lesion and surgical outcome (*χ* ^2^(1) = 50.870, *p* = 0.024), with observed frequencies of seizure‐free outcomes being higher than expected for mesial sclerosis and glioma. Use of intraoperative ECOG was also favorably associated with seizure outcome (*χ* ^2^(1) = 23.784, *p*‐value <0.001), with higher than expected counts in the seizure‐free group.

Regarding temporal lobe surgery, no association was found between the type of surgery (amygdalohippocampectomy vs. partial anterior temporal lobectomy + amygdalectomy vs. anterior temporal lobectomy + amygdalohippocampectomy) and the outcome (Fisher's exact test value was 0.859, *p* = 0.659).

Results of the statistical analyses evaluating potential predictors of long‐term seizure freedom are summarized in Table 4.

**TABLE 4:** Comparison of predictors for seizure‐free vs. non‐seizure‐free outcomes (15‐year follow‐up).

Predictor	Test used	Statistic	*p*‐Value	Conclusion
Gender	Chi‐square	*χ* ^2^(1) = 0.623	0.430	No association
Age	*t*‐test	*M* = 31.91 (SD = 13.00) vs. *M* = 31.99 (SD = 12.52)	0.971	No significant difference
Lesion Localization (Temporal vs. Extra‐Temporal)	Chi‐square	*χ* ^2^(1) = 8.444	<0.05	Temporal lesions associated with better outcome
Lobe Localization	Chi‐square	*χ* ^2^(1) = 14.390	<0.013	Temporal and frontal lesions associated with better outcomes
Laterality	Chi‐square	*χ* ^2^(1) = 6.436	0.04	Left‐sided lesions associated with better outcomes
Type of Lesion	Chi‐square	*χ* ^2^(1) = 50.870	0.024	Better outcomes with mesial sclerosis and glioma
Intraoperative ECOG	Chi‐square	*χ* ^2^(1) = 23.784	<0.001	Use of ECOG associated with better outcomes
Temporal lobe surgeries	Fisher's exact test	Fisher's value = 0.859	0.659	No association

## 4 DISCUSSION

The objective of this study was to characterize a population of patients who underwent epilepsy surgery with at least 15 years of follow‐up. We selected a minimum follow‐up period of 15 years to specifically capture the long‐term durability and evolution of surgical outcomes, which shorter follow‐up intervals may fail to detect. While shorter studies are useful for early postoperative assessment, they may overestimate success by not accounting for late seizure recurrence or changes in antiepileptic drug dependency. By requiring at least 15 years of follow‐up, our study aims to provide a more definitive and realistic picture of epilepsy surgery's lasting impact, addressing important gaps left by shorter‐term research.

Several studies have identified predictors of seizure freedom following epilepsy surgery, most commonly assessed at 1 to 2‐year follow‐up intervals. Factors such as the type of surgery (e.g., temporal lobectomy vs. lesionectomy), operated lobe, etiology of epilepsy, and use of intraoperative electrocorticography seem to have a statistically significant impact on the surgical outcome. Téllez‐Zenteno et al.^5^ conducted a comprehensive meta‐analysis highlighting duration of follow‐up, temporal lobe surgery, and outcome classification system as positive prognostic indicators. Similarly, Mohan et al.^10^ and De Tisi et al.^11^ emphasized the impact of pathology, localization, and completeness of resection on early surgical success. While most of these findings are based on short to mid‐term follow‐up, we hypothesized that the same variables may also influence long‐term outcomes, particularly as seizure recurrence can occur many years after initially successful surgery. Our motivation, therefore, was to re‐evaluate these well‐established predictors in a cohort with at least 15 years of follow‐up, aiming to better understand the durability of surgical benefit over time and to identify which factors remain relevant beyond the typical follow‐up periods reported in the literature.

A relatively small number of patients who had surgery between 1997 and 2009 are still being followed at our referral center. This may be due to several factors: favorable outcomes leading some patients to receive care solely in primary healthcare settings, loss to follow‐up due to missed appointments by those who chose not to resume follow‐up appointments, patients being followed in other healthcare institutions or private clinicians, lack of resources, or death (only 9 patients). Age was not associated with seizure outcome, as has been demonstrated by other researchers. The normal distribution of age in our sample helps to explain this finding, which has already been corroborated by other researchers.^12^, ^13^ Similarly, no differences in seizure outcome were found between males and females. Our sample reveals a percentage of seizure‐free patients of 57.5% at 15 years. These outcomes are somewhat better than previously reported in the literature.^10^, ^14^ However, such studies are scarce and heterogeneous. The use of different scales (sometimes author‐defined) depending on the strict definition of seizure‐free leads to inconsistent results. In a meta‐analysis by Téllez‐Zenteno et al.,^5^ the definition of seizure freedom varied significantly across studies, with some considering patients seizure‐free only if completely free of all seizures without reference to ASMs, while others considered patients seizure‐free if they were free from the most disabling type of seizure. We considered all patients classified as Engel Class I as being seizure free, including those with non‐disabling prodromal symptoms only (I‐B), seizure‐free for the last 2 years (I‐C), or convulsions with ASM discontinuation only (I‐D). In the strictest definition, considering only patients with an Engel I‐A score, the rate would be 31.3%. We opted for the broader definition as it is more easily comparable with most studies. One limitation of this definition is that it does not account for the strategy of tapering ASMs. In our center, tapering is slow and cautious, so a quarter of all patients continue with the same number of ASMs, although they have at least reduced the dose of one of those drugs. The lack of evidence regarding the tapering velocity contributes to the heterogeneity of post‐surgery epilepsy treatment strategies.^15^

Our sample confirms that temporal lobe epilepsy is the most commonly referred type of epilepsy for surgery, with mesial sclerosis emerging as the main etiology, which was significantly underdiagnosed until a few years ago.^5^, ^16^, ^17^ Combining the different results, the patients with the highest probability of seizure freedom are those with left temporal mesial sclerosis. Lateralization, the cerebral lobe involved, and the type of lesion may, therefore, play a role in predicting surgical outcomes.^5^

Interestingly, our results showed a higher frequency of seizure‐free outcomes following left‐sided surgeries, which contrasts with some literature suggesting that resections in the dominant hemisphere (typically the left) are more conservative and thus associated with higher recurrence rates.^18^, ^19^ One possible explanation is that, historically, our surgical team has shown particular caution when approaching lesions on the dominant side, often employing more detailed functional mapping to preserve eloquent cortex. This careful planning may have allowed for more precise resections that maximized seizure control while minimizing neurological risk. Furthermore, a higher prevalence of favorable pathologies—such as mesial temporal sclerosis—in left‐sided cases may have contributed to the better outcomes observed. Although this finding differs from some published reports, it highlights the importance of individualized surgical strategies and supports the need for further investigation in larger, prospective cohorts.

Our statistical analysis did not reveal differences between amygdalohippocampectomy, partial anterior temporal lobectomy + amygdalectomy, and anterior temporal lobectomy + amygdalohippocampectomy. Although the evidence is not always consistent, we were not the first group to find these results. However, as a limiting factor, our sample includes only a small number of cases of selective amygdalohippocampectomy. In individual cases, limited resection was not associated with worse seizure outcomes, and our study aligns with this conclusion.^11^

At our center, intra‐operative ECOG is used selectively, having been employed in 73.6% of patients who underwent resective surgery. Despite the limitations and drawbacks of the technique, our results affirmed the prognostic significance of ECOG, likely due to its association with complete resection of the epileptogenic zone, a factor that has also been consolidated by other researchers as a key prognostic indicator.^17^ On the other hand, poorer outcomes may arise from the inability to resect the entire epileptogenic area due to its proximity to functionally important cortical areas.^5^

Regarding study limitations, we performed a retrospective observational analysis, which comes with its own set of biases. A significant limitation was the lack of precise information regarding surgery dates for patients who underwent multiple procedures, as some records were unclear and some patients struggled with the exact temporal placement of the procedures. Additionally, we aimed to classify the types of seizures experienced by each patient, but the records from 25 years ago were sometimes inadequate for this purpose. Similarly, due to the retrospective nature of the study, which encompassed more than two decades, we were unable to include advanced imaging studies as well as electroencephalographic data, which would have been valuable for a more comprehensive analysis. A key limitation of this study is that outcomes were assessed at a single time point ≥15 years postoperatively, which precluded the capture of interim changes in seizure status or Engel classification. As a result, we were unable to analyze seizure recurrence patterns over time or distinguish between transient and sustained seizure freedom. Nonetheless, this long‐term outcome snapshot provides meaningful insights into the durability of seizure control following surgery.

In conclusion, and in line with what has been previously reported in the literature, epilepsy surgery yields excellent results and represents a valid alternative in the treatment of refractory epilepsy patients. Even though complete discontinuation of ASM is not always achieved, a considerable proportion of patients were seizure‐free in the long term. These outcomes are only achievable through the collaboration of a highly skilled multidisciplinary team that can efficiently integrate every step of the process, from diagnosis and ASM selection to early referral to a specialized refractory epilepsy center, backed by a neurosurgical team with deep expertise in this area.

## CONFLICT OF INTEREST STATEMENT

The authors declare no conflicts of interest.

**Test yourself**

1. Regarding drug‐resistant epilepsy:
  - It is defined as failure to achieve seizure control after one appropriate anti‐seizure medication (ASM) has been tried
  - Less than 10% of patients meet the definition for drug resistance
  - Epilepsy surgery can be an option for these patients, and is possible for both temporal and extratemporal epilepsies
  - Epilepsy surgery is usually not a treatment option for patients with drug‐resistant epilepsy
2. Regarding outcomes after epilepsy surgery in the presented study:
  - The patients with the highest probability of seizure freedom were the ones with left mesial sclerosis
  - The various epilepsy etiologies were not statistically associated with surgical outcomes
  - The best outcomes were statistically associated with right frontal epilepsies
  - All the above options are incorrect
3. Regarding anti‐seizure medication (ASM) use after epilepsy surgery in the presented study:
  - The vast majority of patients were able to stop all ASMs
  - Less than 10% of patients maintained the same ASM and in the same doses
  - More than 60% of patients were able to stop at least one ASM
  - Options B and C are correct

*Answers may be found in the* *supporting information*.

## Supporting information


Data S1.

## Data Availability

The data that support the findings of this study are available on request from the corresponding author. The data are not publicly available due to privacy or ethical restrictions.
