# Supplementary material for: Long‐term outcomes of epilepsy surgery: A 25‐year experience from a tertiary referral center
Source: Epileptic Disord. 2025 Sep 6;27(6):1217–26. doi: 10.1002/epd2.70101 (PMC12747684; doi:10.1002/epd2.70101)
Supplement: Supplementary file 1 — Data S1. [file EPD2-27-1217-s001.docx]

Answers

1. C

2. A

3. D
